# Medical Classes in Philadelphia

**Published:** 1845-12

**Authors:** 


### MEDICAL CLASSES IN PHILADELPHIA.

We are no admirers of vain boasting, nor are we much in the practice of assuming the garb of the prophet, but, judging from the indications around us at the time, we ventured in our last number to express the belief that the number of Medical Students in Philadelphia would be greater the present winter than on any former occasion. What was prophecy then is history now. We constantly hear it said by physicians who go round to the different schools that there cannot be less than a thousand or eleven hundred students in attendance on the lectures. In the Jefferson Medical College, at the earliest period of the course, it was deemed advisable to enlarge the accommodations, and accordingly, additional seats were erected in each of the lecture rooms, and events have shown that the precaution was necessary, for every place is occupied.

The November number of the Western Journal speaks of the prospect of a large class at the Louisville Medical Institute also the present season,

